# Lipid droplet accumulation in β cells in patients with type 2 diabetes is associated with insulin resistance, hyperglycemia and β cell dysfunction involving decreased insulin granules

**DOI:** 10.3389/fendo.2022.996716

**Published:** 2022-09-20

**Authors:** Tomomi Horii, Junji Kozawa, Yukari Fujita, Satoshi Kawata, Harutoshi Ozawa, Chisaki Ishibashi, Sho Yoneda, Takao Nammo, Jun-ichiro Miyagawa, Hidetoshi Eguchi, Iichiro Shimomura

**Affiliations:** ^1^ Department of Metabolic Medicine, Graduate School of Medicine, Osaka University, Suita, Japan; ^2^ Department of Diabetes Care Medicine, Graduate School of Medicine, Osaka University, Suita, Japan; ^3^ Department of Community Medicine, Graduate School of Medicine, Osaka University, Suita, Japan; ^4^ Department of Lifestyle Medicine, Graduate School of Medicine, Osaka University, Suita, Japan; ^5^ Yoneda Clinic, Osaka, Japan; ^6^ Keiseikai Medical Corporation, Osaka, Japan; ^7^ Department of Gastroenterological Surgery, Graduate School of Medicine, Osaka University, Suita, Japan

**Keywords:** Pancreatic fat, lipid droplets, β cell, Type 2 diabetes, ectopic fat

## Abstract

**Background and objective:**

Pancreatic fat is a form of ectopic fat. Lipid droplets (LDs) are also observed in β cells; however, the pathophysiological significance, especially for β cell function, has not been elucidated. Our aim was to assess LD accumulation in β cells in various stages of glucose intolerance and to clarify its relationship with clinical and histological parameters.

**Methods:**

We examined 42 Japanese patients who underwent pancreatectomy. The BODIPY493/503-positive (BODIPY-positive) area in β cells was measured in pancreatic sections from 32 patients. The insulin granule numbers were counted in an additional 10 patients using electron microscopy.

**Results:**

The BODIPY-positive area in β cells in preexisting type 2 diabetes patients was higher than that in normal glucose tolerance patients (p = 0.031). The BODIPY-positive area in β cells was positively correlated with age (r = 0.45, p = 0.0097), HbA1c (r = 0.38, p = 0.0302), fasting plasma glucose (r = 0.37, p = 0.045), and homeostasis model assessment insulin resistance (r = 0.41, p = 0.049) and negatively correlated with an increase in the C-peptide immunoreactivity level by the glucagon test (r = -0.59, p = 0.018). The ratio of mature insulin granule number to total insulin granule number was reduced in the patients with rich LD accumulation in β cells (p = 0.039).

**Conclusions:**

Type 2 diabetes patients had high LD accumulation in β cells, which was associated with insulin resistance, hyperglycemia, aging and β cell dysfunction involving decreased mature insulin granules.

## Introduction

Type 2 diabetes mellitus is a metabolic disease characterized by hyperglycemia resulting from decreased insulin sensitivity and insulin secretion (1). Chronic glucotoxicity and lipotoxicity are thought to be involved in β cell dysfunction in type 2 diabetes mellitus in rodent models (2) and human patients (3, 4).

Ectopic fat, which results from the deposition of excess lipids in organs such as the liver, heart and skeletal muscle where lipid deposition is normally not found, has attracted attention. Pancreatic fat is another form of ectopic fat that is associated with aging and obesity (5). Pancreatic fat is divided into two main components: adipocyte infiltration (fatty infiltration) and intracellular lipid accumulation in both acinar and islet cells (6, 7). We recently reported that fatty infiltration was associated with obesity, insulin resistance, and islet-associated macrophage infiltration (8), which is regarded as islet inflammation (9). We also reported that pancreatic fatty infiltration was associated with postoperative glucose intolerance after pancreatectomy (10). Considering this information, fatty infiltration may have a pathophysiological role in glucose intolerance. Pancreatic fat can also be evaluated by imaging techniques such as computed tomography (CT) (7, 11) and 1H-magnetic resonance spectroscopy (MRS) (7, 12). These imaging modalities can indirectly evaluate pancreatic triglyceride content, and some previous studies have reported pathophysiological roles of this fat accumulation in glucose intolerance (11, 12). However, these modalities cannot distinguish between fatty infiltration and intracellular lipid accumulation. This limitation makes it difficult to understand the pathophysiological significance of each fat component.

Lipids are also deposited as lipid droplets (LDs) within cells, which have a hydrophobic core of neutral lipids covered with a phospholipid monolayer and are found ubiquitously in cells (13, 14). Pancreatic acinar and islet cells also have LDs, and triglyceride accumulation in the islets precedes the onset of overt diabetes in obese Zucker diabetic fatty rats (15). In human pancreatic β cells, age-dependent accumulation of LDs was previously reported (16, 17). LDs are enriched in islet cell areas compared with acinar cell areas in donors with type 2 diabetes (17, 18). However, it has not been elucidated whether LD accumulation, especially in human β cells, is related to islet function and glucose tolerance. The aim of this study was to assess the association of LD accumulation in β cells with clinical and histological parameters, including β cell function, using fresh pancreatic tissue samples from humans with various stages of glucose intolerance.

## Methods

### Patients

We enrolled 32 Japanese patients (21 males and 11 females) who had undergone pancreatic resection from 2008 to 2013 and 10 patients from 2020 to 2021 at the Department of Gastroenterological Surgery, Osaka University Hospital, and agreed to participate in this study. Patients with renal failure (estimated glomerular filtration rate < 30 mL/min/1.73 m2) and patients with pancreatic endocrine tumors were excluded from this study. Patients enrolled from 2008 to 2013 underwent a 75-g oral glucose tolerance test (OGTT) 1-60 days before pancreatic resection, and the results of the test were used to classify the patients into three groups according to diagnostic criteria: normal glucose tolerance (NGT), impaired glucose tolerance (IGT), and newly diagnosed type 2 diabetes mellitus (new T2DM) (19). The classification into a group with preexisting type 2 diabetes mellitus (preexisting T2DM) was based on the clinical history. The patients enrolled after 2020 were classified as non-DM and T2DM based on their medical history, fasting plasma glucose and HbA1c (19).

### Laboratory tests

We evaluated HbA1c (%, mmol/mol), fasting plasma glucose (FPG) (mmol/L), fasting C-peptide immunoreactivity (F-CPR) (nmol/L), fasting immunoreactive insulin (F-IRI) (pmol/L), the C-peptide index (CPI) (nmol/mmol), the insulinogenic index (I.I.) (pmol/mmol), homeostasis model assessment insulin resistance (HOMA-IR), total cholesterol (mg/dl), triglycerides (mg/dl) and ΔC-peptide (ΔCPR) (nmol/L). The CPI, which is an indicator of the basal insulin secretory capacity, was calculated by F-CPR (nmol/L)/FPG (mmol/L). The insulinogenic index, which is an indicator of bolus insulin capacity, was defined as the ratio of the increment in plasma insulin level to that of the plasma glucose level at 30 min during the 75 g-OGTT [Δserum insulin 0-30 (pmol/L)/Δplasma glucose 0-30 min (mmol/L)]. The value of ΔC-peptide was defined as the increment in serum C-peptide level (nmol/L) at 6 min after intravenous injection of 1 mg of glucagon after an overnight fast.

### Pancreatic tissue processing

We obtained 32 normal pancreatic frozen and paraffin tissue samples from patients who had undergone pancreatic resection from 2008 to 2013. The tissues were isolated near the resected margins after intraoperative consultation and were divided into frozen and paraffin samples. The tissues were frozen immediately in liquid nitrogen after being embedded in optimal cutting temperature compound (Sakura Finetek USA, Torrance, California). Sequential 5-µm-thick sections were cut on a cryostat, confirmed to be noncancerous by hematoxylin and eosin (HE) staining, and then stored at -80°C until use. Other tissues were also fixed immediately in formaldehyde and embedded in paraffin, and 5-μm-thick sections were cut for subsequent analysis. Paraffin-embedded tissue was stained with HE and confirmed to be noncancerous. Sections with a >30% fibrous area estimated by Azan staining using paraffin samples were excluded from this study.

### Electron microscopic evaluation

We obtained 10 normal pancreatic tissue samples from patients who underwent pancreatic resection from 2020 to 2021 for electron microscopic evaluation. Pancreatic tissues were fixed with 2.5% glutaraldehyde for 2 hours. After fixation with 1% osmium tetroxide for 90 min, the cells were dehydrated through a graded series of ethanol (50%-100%) and propylene oxide. Finally, they were embedded in epoxy resin. Ultrathin sections were cut on an ultramicrotome (Ultracut E; Reichert-Jung, Vienna, Austria), stained with uranyl acetate and lead citrate and observed by transmission electron microscopy (Hitachi HT7800, Hitachi High-Tech Corporation, Tokyo, Japan). We counted LD numbers in β cells and evaluated them as LD numbers per unit β cell. We also manually counted the total insulin granule numbers per unit β cell area (/μm2), the mature insulin granule numbers per unit β cell area (/μm2) and the ratio of the mature insulin granule numbers to the total insulin granule numbers (%). The β cell area was calculated by excluding the nuclear area. Mature insulin granules were defined by the presence of a halo and core density (20). We also counted the number of autophagic vacuoles per unit β cell. Autophagic vacuoles were identified by an intracellular double membrane structure containing cytoplasmic material and/or massive vacuolization (21). We examined 10 randomly selected β cells per patient.

### Immunohistochemistry

The primary and secondary antibodies are listed in **Table S1** in Supplementary Material. To evaluate LDs in β cells, we used double immunofluorescence staining of frozen samples. β cells were labeled with C-peptide, and LDs were labeled with BODIPY 493/503. First, the sections were incubated with mouse anti-C-peptide antibody and goat anti-mouse Alexa Fluor 594-conjugated secondary antibody. The same section was then incubated with 10 μmol/L BODIPY. The ratio of the sum of the BODIPY-positive area to the entire β cell area was defined as the “BODIPY-positive area in β cells” (%), and LD area was assessed by BODIPY-positive area. We observed 18.5 ± 19.1 islets per section. A mean section area of 9.4 ± 6.6 mm2 was examined for each section. Images were acquired on a confocal laser scanning microscope (FV1200 IX81 OLYMPUS, Tokyo, Japan) and measured using WinROOF software (Mitani Corporation, Fukui, Japan).

To evaluate intra- and peripheral islet macrophages, we performed double immunofluorescent staining for insulin and CD68, which is a marker of macrophages, in the same way as in a previous report with paraffin samples (8). We counted the number of CD68-positive (CD68+) cells per islet, which was defined as CD68+ cells around the periphery and/or within islets of more than 100 μm in diameter and expressed in units of the number per islet. We observed 76.7 ± 70.5 islets per section. Images were acquired on a fluorescence microscope (BX53; Olympus, Tokyo, Japan).

To evaluate the relative β cell area compared with the entire pancreatic section, we performed the indirect immunoperoxidase technique in the same way as in previous reports with paraffin samples (22, 23). Images were acquired on a fluorescence microscope (BX53; Olympus, Tokyo, Japan).

### Assessment of pancreatic fatty infiltration

Pancreatic fatty infiltration was evaluated using paraffin-embedded tissue stained with HE. The ratio of the sum of the interlobular and intralobular fat-cell area to the entire pancreatic section was defined as the “fat-cell area” (%) (24). A mean section area of 63.1 ± 41.2 mm2 was examined for each section. Images were acquired on an optical microscope and quantified using the WinROOF software program (10).

### Statistical analysis

Normally distributed data are presented as the means ± SDs, and nonnormally distributed data are presented as the medians and interquartile ranges (IQRs). Groups of data with a normal distribution were compared using one-way analysis of variance followed by a post hoc Tukey–Kramer analysis. For data that were not normally distributed, the Kruskal–Wallis test followed by a post hoc Steel-Dwass analysis was used. Significance was set at p < 0.05. All statistical analyses were performed with JMP Pro 14 software (Statistical Analysis System Inc. Cary, NC, USA).

## Results

### Clinical characteristics and laboratory data

**Table 1** shows a comparison of the clinical characteristics among the groups of 32 patients whose LDs in β cells were examined by BODIPY staining. Patients were classified into the NGT (n = 9), IGT (n = 8), new T2DM (n = 6), or preexisting T2DM (n = 9) groups. Primary diseases were mainly cystic lesions of the pancreas (n = 11) and pancreatic cancer (n = 12). The surgical procedures were pancreatoduodenectomy (PD) (n = 21), distal pancreatectomy (DP) (n = 10) or total pancreatectomy (n = 1). There were no differences among the groups with respect to BMI, F-IRI, I.I., total cholesterol or triglycerides. Age was higher in the preexisting T2DM group than in the NGT group (p < 0.05). HbA1c levels were higher in the new T2DM group than in the NGT group (p < 0.05) and were higher in the preexisting T2DM group than in the NGT (p < 0.01), IGT (p < 0.01) and new T2DM (p < 0.05) groups. The FPG level in the preexisting T2DM group was higher than that in the NGT (p < 0.01), IGT (p < 0.01) and new T2DM (p < 0.05) groups. The F-CPR level was lower in the preexisting T2DM group than in the new T2DM group (p < 0.05). The CPI was lower in the preexisting T2DM group than in the new T2DM group (p < 0.05).

**Table 1:** Clinical Characteristics of 32 Subjects.

	Total	NGT	IGT	New T2DM	Preexisting T2DM
N	32	9	8	6	9
Male/female	21/11	7/2	4/4	4/2	6/3
Age, y	67.3 ± 10.6	59.3 ± 12.3	69.8 ± 8.3	65.3 ± 8.3	74.2 ± 6.8^*^
BMI, kg/m^2^	21.4 ± 2.8	21.0 ± 2.4	22.6 ± 3.4	22.8 ± 1.9	19.9 ± 2.7
HbA1c (%, mmol/mol)	6.1 ± 1.1,43.6 ± 12.6	5.2 ± 0.6,33.7 ± 6.6	5.5 ± 0.3,36.3 ± 3.8	6.3 ± 0.5^*^,45.3 ± 5.3	7.5 ± 1.0^**††‡^58.6 ± 11.0
FPG, mmol/L	5.8 ± 1.1 (n=30)	5.3 ± 0.3	5.2 ± 0.4	5.9 ± 1.1	7.2 ± 1.2^**††‡^(n=7)
F-IRI, pmol/L	36.2 ± 18.8 (n=24)	33.4 ± 13.8	33.7 ± 17.5	46.8 ± 25.9	18.2 (n=1)
F-CPR, nmol/L	0.5 ± 0.2 (n=27)	0.5 ± 0.1	0.5 ± 0.2	0.6 ± 0.3	0.2 ± 0.05^‡^ (n=4)
CPI, nmol/mmol	9.1 ± 4.2 (n=27)	10.3 ± 2.2	9.2 ± 4.2	10.6 ± 5.4	3.6 ± 0.9^‡^ (n=4)
Insulinogenic index, pmol/mmol	92.3 ± 95.7 (n=23)	108.7 ± 61.9	125.2 ± 136.3	27.3 ± 15.8 (n=5)	6.6 (n=1)
ΔCPR, nmol/L	0.9 ± 0.4 (n=15)	1.2 ± 0.2 (n=6)	1.0 ± 0.3 (n=4)	0.7 ± 0.1 (n=2)	0.3 ± 0.1^**††^ (n=3)
HOMA-IR	0.5 ± 0.3 (n=24)	0.4 ± 0.2	0.4 ± 0.2	0.7 ± 0.4	0.3 (n=1)
Total cholesterol,mg/dL	196.5 ± 32.1 (n=27)	183.0 ± 41.7 (n=7)	210.7 ± 21.4 (n=7)	197.2 ± 25.8 (n=5)	195.4 ± 34.6 (n=8)
Triglycerides, mg/dl	113.3 ± 45.5 (n=29)	138.9 ± 56.6 (n=8)	117.3 ± 39.9	103.4 ± 46.3 (n=5)	90.1 ± 28.1 (n=8)
Fat-cell area, %	2.2 ± 2.6	2.0 ± 2.3	2.6 ± 2.7	1.9 ± 3.7	2.4 ± 2.5
Relative β cell area, %	0.9 ± 0.4	1.0 ± 0.5	1.0 ± 0.5	0.8 ± 0.5	0.8 ± 0.3
CD68^+^ cells/islet	0.04 ± 0.06	0.01 ± 0.02	0.01 ± 0.01	0.07 ± 0.09	0.08 ± 0.06
Medication for diabetes		–	–		None: 1 SU: 5, αGI: 2, Insulin: 3
Underlying disease	Pancreatic cancer: 12IPMN: 11Serous cystic adenoma: 1 Bile duct cancer: 5 Carcinoma of the papilla of Vater: 1 Pseudocyst of pancreas: 1 Pancreatic metastasis from renal cell carcinoma: 1	Pancreatic cancer: 3 IPMN: 2 Bile duct cancer: 3 Pseudocyst of pancreas: 1	Pancreatic cancer: 5 IPMN: 2 Bile duct cancer: 1	Pancreatic cancer: 1 IPMN: 4 Carcinoma of the papilla of Vater: 1	Pancreatic cancer: 3 IPMN: 3 Serous cystic adenoma: 1 Bile duct cancer: 1 Pancreatic metastasis from renal cell carcinoma: 1

Data are expressed as the mean ± SD. ^*^p<0.05 vs. NGT, ^**^p<0.01 vs. NGT, ^†^p<0.05 vs. IGT, ^††^p<0.01 vs. IGT, ^‡^p<0.05 vs. New T2DM, ^‡‡^p<0.01 vs. New T2DM. Abbreviations: BMI, body mass index; FPG, fasting plasma glucose; F-IRI, fasting immunoreactive insulin; F-CPR, fasting C-peptide; CPI (C-peptide index), fasting C-peptide/fasting plasma glucose; Insulinogenic index, ΔIRI [IRI 30 minutes-IRI 0 minutes]/ΔPG [PG 30 minutes-PG 0 minutes]; ΔCPR (ΔC-peptide), the increment in serum C-peptide level at 6 min after intravenous injection of 1 mg of glucagon after an overnight fast; HOMA-IR, homeostasis model assessment of insulin resistance; IPMN, intraductal papillary mucinous neoplasm; SU, sulfonylurea; αGI, alpha-glucosidase inhibitor.

### Relationships between lipid droplet accumulation in β cells and glucose tolerance

**Figure 1:**
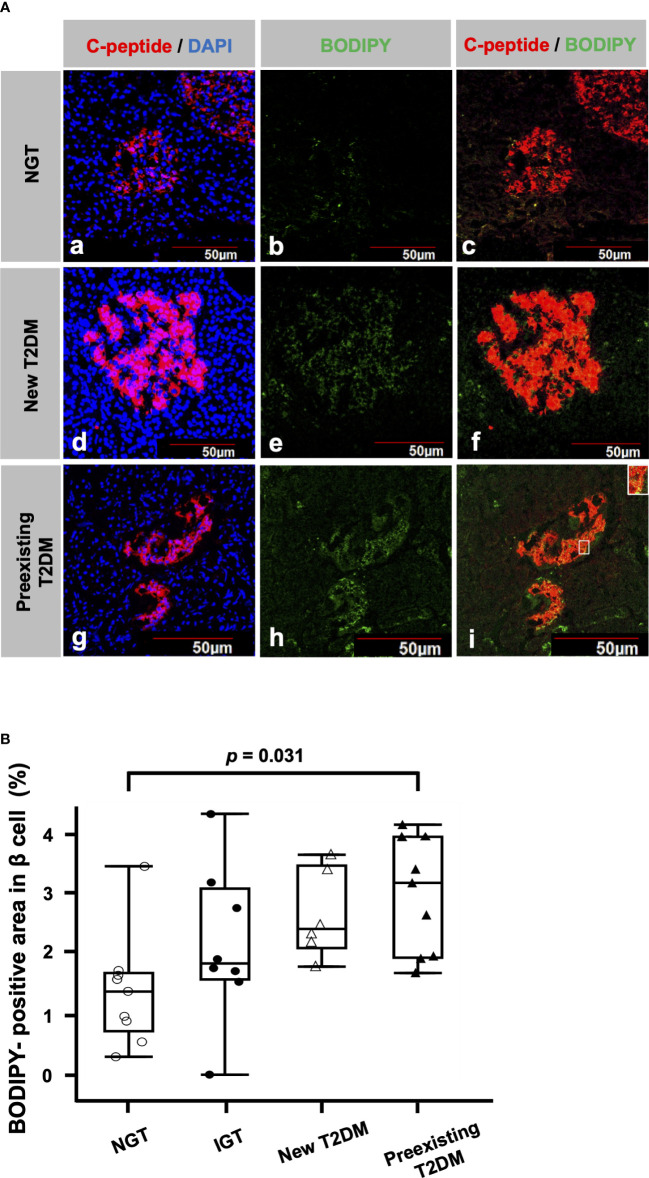
Lipid droplets (LDs) in β cells **(A)** Representative immunofluorescence staining for C-peptide (red), BODIPY (green) and DAPI (blue) from patients with NGT **(a-c)**, newly diagnosed T2DM **(d-f)** and preexisting T2DM **(g-i)**. Bars, 50 μm. **(B)**: Comparisons of BODIPY-positive areas in β cells among the NGT (n= 9), IGT (n= 8), newly diagnosed T2DM (n= 6) and preexisting T2DM groups (n= 9).

**Figure 1** shows a representative image of double immunofluorescence staining for C-peptide and BODIPY. The BODIPY-positive areas in the β cells of patients with new T2DM (**Figure 1A (d-f)**) and preexisting T2DM (**Figure 1A (g-i)**) were larger than those of a patient with NGT (**Figure 1A (a-c)**). **Figure 1B** shows a comparison of BODIPY-positive areas in β cells among the patients with NGT, IGT, new T2DM and preexisting T2DM. The median BODIPY-positive area in β cells was 1.4% (IQR, 0.97%), 1.9% (IQR, 1.5%), 2.4% (IQR, 1.4%) and 3.2% (IQR, 2.0%) in the NGT, IGT, new T2DM and preexisting T2DM groups, respectively. The BODIPY-positive area in β cells in the preexisting T2DM group was higher than that in the NGT group (p = 0.031). The median BODIPY-positive area in β cells in the preexisting T2DM group still tended to be higher than that in the NGT group even after age adjustment (p = 0.057).

### Relationships between lipid droplet accumulation in β cells or acinar cells and surgical procedures

We examined BODIPY-positive areas of the two groups; the PD group and the DP group. The BODIPY-positive area in the PD group represents that in head or uncus of the pancreas, while the BODIPY-positive area in the DP group represents that in body or tail of the pancreas. There were no significant differences in BODIPY-positive areas in β cells between the groups (the PD group; 2.43 ± 0.24% vs the DP group; 2.23 ± 0.35%, p = 0.64). It seems that the BODIPY-positive area in the acinar area was low compared to that in the islet area both in the PD group and the DP group.

### Correlations between lipid droplet accumulation in β cells and various parameters

**Figure 2:**
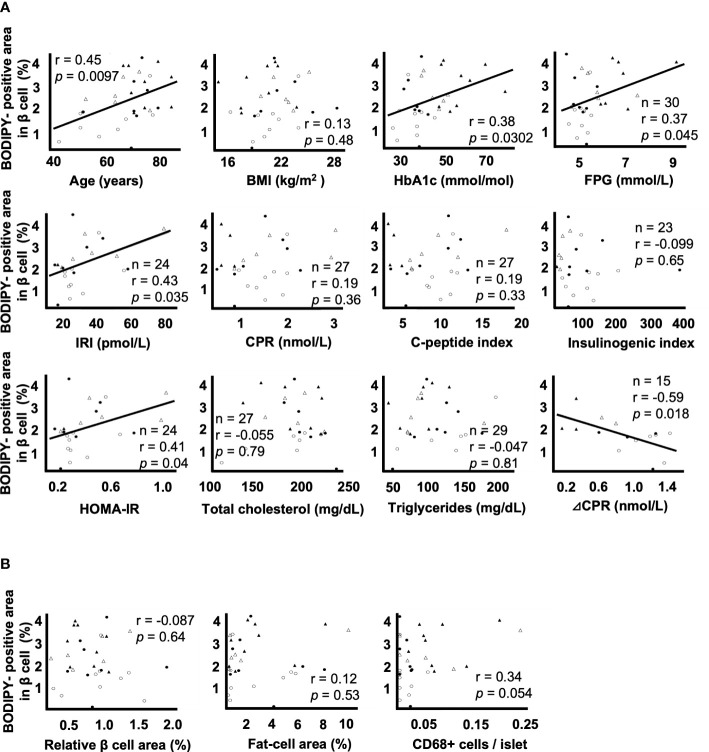
Correlation coefficients between lipid droplets (LDs) in β cells and clinical and histological parameters **A**: Correlation coefficients between the BODIPY-positive area in β cells and the parameters of age, BMI, HbA1c, FPG, F-IRI, F-CPR, C-peptide index, insulinogenic index, HOMA-IR, total cholesterol, triglycerides and ΔCPR. **B**: Correlation coefficients between the BODIPY-positive area in β cells and the relative β cell area, fat-cell area and CD68^+^ cells per islet. Open circles (○), the NGT group (n= 9); closed circles (●), the IGT group (n= 8); open triangles (∆), the newly diagnosed T2DM group (n= 6); closed triangles (▲), the preexisting T2DM group (n= 9).

**Figure 2A** shows the correlation between the BODIPY-positive area in β cells and clinical parameters. BODIPY-positive area in β cells was associated with age (r = 0.45, p = 0.0097), HbA1c (r = 0.38, p = 0.0302), FPG (r = 0.37, p = 0.045), F-IRI (r = 0.43, p = 0.035), HOMA-IR (r = 0.41, p = 0.049) and ΔCPR (r = -0.59, p = 0.018) but not with BMI (r = 0.13, p = 0.48), F-CPR (r = 0.19, p = 0.36), CPI (r = 0.19, p = 0.33), I.I. (r = -0.099, p = 0.65), total cholesterol (r = -0.055, p = 0.79) or triglycerides (r = -0.047, p = 0.81). Since the maximum sample size was 32, multiple regression analyses were performed using the two explanatory variables; the age and the other clinical parameters. IRI (p = 0.0020) and HOMA-IR (p = 0.0027) were independently associated, and FPG (p = 0.066) tended to be associated with BODIPY-positive area in β cells. Due to the strong correlation (multicollinearity) between age and ΔCPR (r = -0.75 p = 0.0012), the multiple regression analysis could not be done.

**Figure 2B** shows the correlation between the BODIPY-positive area in β cells and histological evaluation. The BODIPY-positive area in β cells was not associated with the relative β cell area (r = -0.087, p = 0.64), fat-cell area (r = 0.12, p = 0.53) or the number of CD68+ cells per islet (r = 0.34, p = 0.054).

### Lipid droplet accumulation in β cells by electron microscopy

To investigate the association between LD accumulation in β cells and the endogenous insulin secretory capacity, we investigated the ultrastructural changes in β cells by electron microscopy. **Table S2** in Supplementary Material shows the clinical characteristics of the groups. Patients were classified as non-DM (n = 6) and T2DM (n = 4). There were no differences between the groups with respect to age, BMI or FPG. HbA1c levels were higher in the T2DM group than in the non-DM group (p < 0.05).

**Figure 3:**
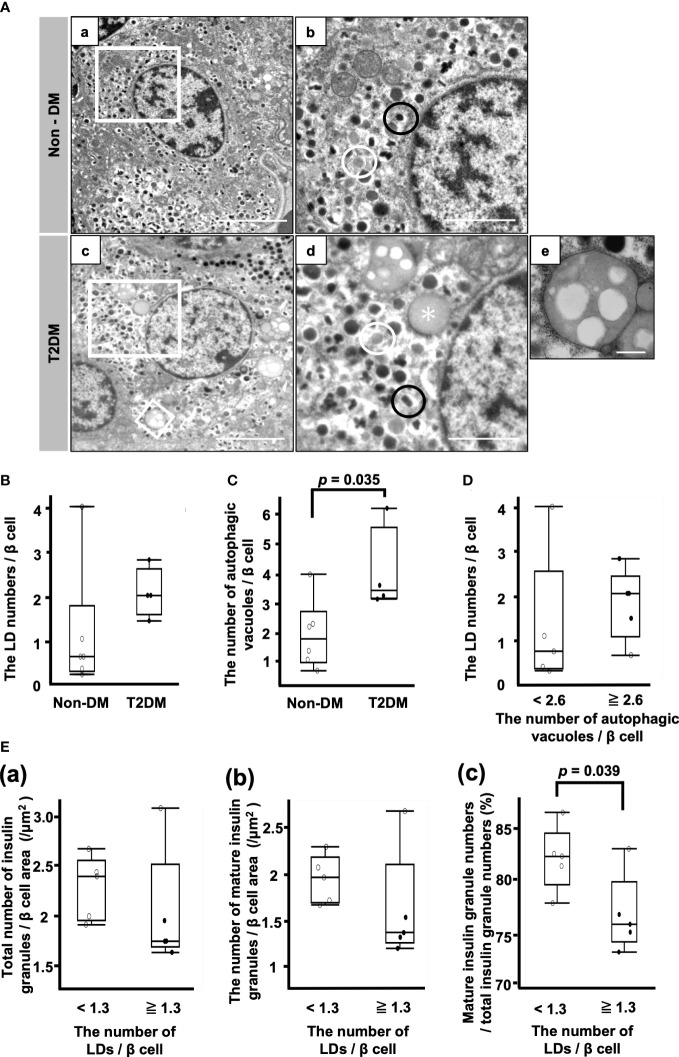
Analysis of β cells by electron microscopy. **(A)** Representative electron microscopy images of β cells from patients with non-DM **(a, b)** and T2DM **(c–e)**. **(b, d)**: Magnification of the white squares in **(a, c)**, respectively. **(e)**: Autophagic vacuoles. Magnification of the smaller white square in **(c)**. Black circle, mature insulin granule; white circle, immature insulin granule; asterisk, lipid droplet. Bars: a and c, 5 μm; b and d, 2 μm; e, 500 nm. **(B)** The LD numbers per β cell. **(C)** Autophagic vacuole numbers per β cell. **(D)** Comparison of LD numbers per β cell between the two groups of patients whose autophagic vacuole numbers per β cell were equal to or more than 2.6/β cell or less than 2.6/β cell. **(E)** Comparison of the total number of insulin granules **(a)**, the number of mature insulin granules **(b)** and the ratio of mature insulin granule number to total insulin granule number **(c)** between the two groups of patients whose LD numbers per β cell were equal to or more than 1.3/β cell or less than 1.3/β cell. Open circles (○), the Non-DM group (n=6); closed circles (●), the T2DM group (n=4).

**Figure 3A** shows representative electron microscopic images of β cells. Different kinds of insulin granules are seen in both patients with non-DM and T2DM, and lipid droplets and autophagic vacuoles are especially common in patients with T2DM (**Figure 3A (d, e)**).

There was no difference between the non-DM and T2DM groups with respect to the LD numbers/β cell (**Figure 3B**). The number of autophagic vacuoles was significantly increased in the T2DM groups (p = 0.035) (**Figure 3C**). Next, we divided 10 patients into the high autophagic vacuole number group and the low autophagic vacuole number group by the median autophagic vacuole numbers/β cell (2.6/β cell) and compared the LD numbers/β cell between the groups (**Figure 3D**). There was no difference between them, while the median value of the LD numbers/β cell in the high autophagic vacuole number group was higher than that in the low autophagic vacuole number group.

**Figure 3E** shows the comparison of the insulin granule numbers between the two groups of patients whose LD numbers/β cell were more or less than the median of 1.3. There were no differences between the groups with respect to the total number of insulin granules and the number of mature insulin granules/β cell area (**Figure 3E (a, b)**). However, the ratio of mature insulin granule number to total insulin granule number was reduced in the high LD group (**Figure 3E (c)**). The median ratio of mature insulin granule number to total insulin granule number in the low LD group was 82.5% (IQR, 5.1%) and that of the high LD group was 75.8% (IQR, 6.0%) (p = 0.039) (**Figure 3E (c)**).

## Discussion

The present study demonstrated that LDs in β cells accumulated more in patients with type 2 diabetes than in normal glucose-tolerant subjects and that LD accumulation was associated with insulin resistance, hyperglycemia and decreased insulin secretion. We also revealed that LD accumulation was associated with decreased mature insulin granules in an ultrastructural analysis. LDs in β cells have been known to be associated only with aging until now. This is the first study that clearly showed, using fresh tissue samples from humans, that LD accumulation in β cells was associated with diabetic pathophysiological conditions as well as type 2 diabetes.

Type 2 diabetes is characterized by insulin resistance (25), which could lead to an increase in free fatty acids (FFAs) derived from triglyceride degradation (26), and FFAs may flow into β cells. In addition, the BODIPY-positive area in β cells was associated with HbA1c and FPG in this study. It was reported that primary human islets treated with FFAs and hyperglycemia showed the increases of the size and the number of intracellular LDs in time- and concentration-dependent manner (27). Lipid deposition in β cells was also increased in response to high-fat diet in animal model transplanted human islets (28). The metabolisms of both FFAs and glucose in β cells are linked through the glycerolipid/FFA cycle, and LDs are produced in that process (29), suggesting that hyperglycemia in addition to excess FFAs in a state of insulin resistance are related to LD accumulation, especially in type 2 diabetes patients.

Both neutral lipase including adipose triglyceride lipase (ATGL) and lysosomal acid lipase (lipophagy) mediate LD degradation (30), while the extent of contribution of lipophagy to LD degradation is still unclear (31, 32). ATGL-mediated lipolysis is important in lipid homeostasis and insulin secretion (29). In fact, downregulation of ATGL increased LD in human β cell and impaired insulin secretion (33). In contrast, it is reported that the role of autophagy is to prompt lipid buildup in LDs by replenishing LDs with new FFAs (34). Thus, LD formation is closely associated with autophagy which has been shown to be markedly increased in type 2 diabetes patients (35, 36). In fact, autophagic vacuoles were significantly increased in type 2 diabetes patients in this study, though it is inconclusive whether these data indicate enhanced autophagic activity or blockade of autophagy (37). It may be postulated that LDs also accumulate at least due to the alteration of autophagy.

LD accumulation in β cells was associated with decreased insulin secretory capacity and decreased mature insulin granules. Downregulation of perilipin 2 (PLIN2), which is a key scaffold protein and resides on the surface of LDs, ameliorates the effects of fatty acid- and chemical-induced ER stress, whereas PLIN2 overexpression exacerbates them (38). Contrary to that report, the other studies showed that downregulation of PLIN2 reduced the levels of FFAs incorporated into LD and resulted in mitochondrial dysfunction (39) and endoplasmic reticulum (ER) stress (40), as LD formation protects cells against toxic effects of FFA (41). In any case, excess LD accumulation may reflect inappropriate lipid metabolism beyond FFA homeostasis, which causes lipotoxicity-induced endoplasmic reticulum stress, resulting in impaired insulin granule maturation and decreased insulin secretory capacity (42, 43). In addition, considering the association of LD formation and autophagy as mentioned previously, LD accumulation may be accelerated by altered autophagy of immature or old insulin granules in type 2 diabetes (35), probably resulting in decreased mature insulin granules.

There are several limitations that should be considered when interpreting the results of this study. First, BODIPY493/503 represents the mixture of LDs and lipofuscins (31), which are known to increase with aging and quite abundant in adult β cells (44). This might be a reason that LDs detected by electron microscopy did not show a difference between the non-DM and T2DM groups, though BODIPY-positive area was increased in T2DM patients who were older and expected to have more lipofuscins in beta cells. Second, there are no data about serum FFA levels. Although data about total cholesterol and triglycerides exist, these lipid profiles did not correlate with BODIPY-positive area in β cells, which may be primarily because some patients received antihyperlipidemic drugs, and the lipid profile may not directly reflect intracellular lipid metabolism. Third, the patients had pancreatic diseases, mainly malignant diseases, which might affect lipid deposition in β cells. However, there were no significant differences in BODIPY-positive area in β cells between patients with malignant diseases and those without (data not shown). Finally, the numbers of patients were relatively small, especially in the new T2DM group. Importantly, despite these limitations, we were able to clarify the pathophysiological significance of LDs in β cells based on detailed clinical parameters in conjunction with pancreatic histological analyses using fresh tissue samples from humans.

In conclusion, the present study demonstrated that LDs in β cells were associated with insulin resistance, hyperglycemia and β cell dysfunction involving decreased mature insulin granules in type 2 diabetes.

## Data availability statement

The original contributions presented in the study are included in the article/supplementary material. Further inquiries can be directed to the corresponding author.

## Ethics statement

The studies involving human participants were reviewed and approved by the Ethics Committee of Osaka University. The patients/participants provided their written informed consent to participate in this study.

## Author contributions

TH analyzed the data and wrote the manuscript. YF, SK, HO, CI, SY, and TN contributed to the discussion. HE examined the patients and obtained pancreatic tissue samples. YF and JK analyzed the data and reviewed/edited the manuscript. J-IM and IS contributed to the discussion and reviewed/edited the manuscript. TH is the guarantor of this work and as such, had full access to all the data in the study and takes responsibility for the integrity of the data and the accuracy of data analysis. All authors contributed to the article and approved the submitted version.

## Funding

This study was supported in part by a Grant-in-Aid from Japan Society for the Promotion of Science (Grant Number 19K09024).

## Acknowledgments

We thank Ms. Misako Kobayashi for excellent technical assistance. We are also grateful to Eiji Oiki and Tomoaki Mizuno (Center for Medical Research and Education, Graduate School of Medicine, Osaka University) for technical support with electron microscopy.

## Conflict of interest

J-IM is employed by Keiseikai Medical Corporation, Osaka, Japan.

The remaining authors declare that the research was conducted in the absence of any commercial or financial relationships that could be construed as a potential conflict of interest.

## Publisher’s note

All claims expressed in this article are solely those of the authors and do not necessarily represent those of their affiliated organizations, or those of the publisher, the editors and the reviewers. Any product that may be evaluated in this article, or claim that may be made by its manufacturer, is not guaranteed or endorsed by the publisher.
