# Supplementary material for: Lipid droplet accumulation in β cells in patients with type 2 diabetes is associated with insulin resistance, hyperglycemia and β cell dysfunction involving decreased insulin granules
Source: Front Endocrinol (Lausanne). 2022 Sep 20;13:996716. doi: 10.3389/fendo.2022.996716 (PMC9530467; doi:10.3389/fendo.2022.996716)
Supplement: Supplementary file 1 [file Table_1.docx]

**Supplementary Material**

**Table S1**

Primary antibodies (A), secondary antibodies (B), chromogenic substrates (C) and fluorescent dye (D) used

**Table S2**

Clinical characteristic of 10 subjects whose pancreatic tissue samples were examined by electron microscopy

**Table S1**

Primary antibodies (A), secondary antibodies (B), chromogenic substrates (C) and fluorescent dye (D) used

A. Primary antibodies

| Antigen | Species | Source | RRID | Dilution |
| --- | --- | --- | --- | --- |
| insulin | guinea pig | Dako Japan, Kyoto, Japan | AB_10013624 | 1:400 |
| C-peptide | Mouse | NovoClone; Novo Nordisk, Bags- vaerd, DK | − | 1:100 |
| CD68 | Rabbit | Abcam, Cambridge, UK | AB_10971844 | 1:50 |

B. Secondary antibodies

| Antigen | Species | Source | RRID | Dilution |
| --- | --- | --- | --- | --- |
| guinea pig | goat (Alexa Fluor 555-conjugated) | Thermo Fisher Scientific, OR, USA | AB_2535856 | 1:400 |
| guinea pig | goat (biotinylated) | Vector Laboratories, CA, USA | AB_2336132 | 1:200 |
| mouse | goat (Alexa Fluor 594-conjugated) | Thermo Fisher Scientific, OR, USA | AB_2534091 | 1:200 |
| rabbit | goat (biotinylated) | Vector Laboratories, CA, USA | AB_2313606 | 1:200 |

C. Chromogenic substrates

| Chromogenic substrates | Source |
| --- | --- |
| Avidin-biotin complex (ABC) kit | Vector Laboratories, CA, USA |
| 3,3-diaminobenzidine (DAB) | Molecular Probes, OR, USA |
| Streptavidin, Alexa Fluor 488-conjugated | Invitrogen by Thermo Fisher Scientific |

D. Fluorescent dye

| Fluorescent dye | Source |
| --- | --- |
| 4,4-Difluoro-1,3,5,7,8-Pentamethyl-4-Bora-3a,4a-Diaza-S-Indancene (BODIPY493/503) | Invitrogen by Thermo Fisher Scientific |

**Table S2**

Clinical characteristic of 10 subjects whose pancreatic tissue samples were examined by electron microscopy

|  | Total | Non-DM | T2DM |
| --- | --- | --- | --- |
| n | 10 | 6 | 4 |
| Male/female | 5/5 | 2/4 | 3/1 |
| Age (years) | 68.1±12.5 | 64.3±13.8 | 73.8±9.0 |
| BMI (kg/m^2^) | 23.8±4.2 | 22.8±3.2 | 25.1±5.7 |
| HbA1c  (%, mmol/mol) | 6.2±0.9,  43.9 ±9.6 | 5.7±0.3,  38.6±3.4 | 6.9±1.0^*^,  51.9±10.7 |
| FPG (mmol/L) | 5.3±1.0 (n=8) | 4.9±0.5 (n=5) | 6.1±1.4 (n=3) |
| Medication for diabetes |  | - | TZD: 1, Insulin: 3 |
| Underlying disease | Pancreatic cancer: 6, IPMN: 2, MCN: 2 | Pancreatic cancer: 3, IPMN: 1, MCN: 2 | Pancreatic cancer: 3, IPMN: 1 |

Data are expressed as mean±SD or median (interquartile range)

^*^*p<*0.05 vs. non-DM,

Abbreviations: BMI, body mass index; FPG, fasting plasma glucose; IPMN, Intraductal papillary mucinous neoplasm; MCN, Mucinous cystic neoplasm; TZD, thiazolidinedione
